# A typology of interdisciplinary collaborations: insights from agri-food transformation research

**DOI:** 10.1007/s11625-025-01702-x

**Published:** 2025-06-20

**Authors:** Benjamin Hofmann, Ueli Reber, Priska Ammann, Julia Dötzer, Jennifer Mark, Chloe McCallum, Milena Wiget, Lucca Zachmann

**Affiliations:** 1 Department of Environmental Social Sciences, Eawag: Swiss Federal Institute of Aquatic Science and Technology, Überlandstrasse 133, 8600 Dübendorf, Switzerland; 2 Institute of Political Science, University of Bern, Bern, Switzerland; 3 Swiss Tropical and Public Health Institute, Allschwil, Switzerland; 4 University of Basel, Basel, Switzerland; 5 Research Institute of Organic Agriculture FiBL, Frick, Switzerland; 6 Agricultural Economics and Policy Group, ETH Zurich, Zurich, Switzerland; 7 Queen’s Business School, Queen’s University Belfast, Belfast, UK

**Keywords:** Interdisciplinary research, Research design, Methods, Research collaborations, Sustainability transformation, Agriculture, Reflection

## Abstract

To understand complex societal transformations, scholars have called for more interdisciplinary research in which researchers from various disciplines collaborate. To support the implementation of such collaborations, we introduce a novel typology of interdisciplinary collaborations developed from the literature and from structured reflection on our own research experience. The typology distinguishes (I) common base, (II) common destination, and (III) sequential link type of interdisciplinary collaborations. Common base refers to an interdisciplinary collaboration at one research stage that later separates into parallel disciplinary work; common destination to a collaboration where separate disciplinary work feeds into joint interdisciplinary work at the next stage; and sequential link to a completed stage of disciplinary research that provides the basis for research in another discipline. We illustrate the typology with a case study of interdisciplinary collaborations in a research project that studied the potential for an evidence-based transformation of agricultural pesticide governance. The project involved researchers from seven natural, health, and social science disciplines who developed a process for forming and maintaining interdisciplinary collaborations. We provide five examples of interdisciplinary collaborations from the project, explaining for each its practical design and implementation, its contribution to overall research goals, and related opportunities and challenges. The examples show that the typology can systematize the thinking about interdisciplinary collaborations and enable critical reflection about interdisciplinary research design and implementation. Based on our reflections as early-career researchers, we conclude with lessons that can inform future interdisciplinary research projects on agri-food transformation and beyond.

## Introduction

There is a large and growing literature on sustainability transitions that lead to transformation in different societal sectors, including energy (Lindberg et al. 2019; Vanegas Cantarero 2020), transportation (Dominković et al. 2018; Griffiths et al. 2021), water (Gleick 2018; Pahl-Wostl 2019), urban environments (Bulkeley et al. 2016; Elmqvist et al. 2019), and agri-food systems (Caron et al. 2014; Bui et al. 2016; Vermunt et al. 2020; Barrett et al. 2020). The literature generally shows that transformations are highly complex, as they depend on factors at different levels in policy and practice, including interactions between niche innovation and regulatory regimes (Köhler et al. 2019), as well as changes in values, social structures, system feedbacks, and parameters (Abson et al. 2017). This complex multi-level character implies that disciplinary lenses can usually shed light only on sub-parts of sustainable transformation processes.

Obtaining a more comprehensive picture requires interdisciplinary research, which is “a mode of research by teams or individuals that integrates perspectives/concepts/theories, and/or tools/techniques, and/or information/data from two or more bodies of specialized knowledge or research practice” (Porter 2006, p. 189). Calls for more interdisciplinary research are abundant in sustainability science (Kelly et al. 2019; Pauliuk 2020; Hernandez-Aguilera et al. 2021). In addition, the growing stream of transdisciplinary research, which is characterized by knowledge coproduction involving scientists and societal stakeholders, builds on interdisciplinary perspectives (Brandt et al. 2013). However, the implementation of interdisciplinarity in project-based collaborations of researchers is often challenging (Freeth and Caniglia 2020; Cairns et al. 2020; Deutsch et al. 2021). A persistent challenge is that researchers need to understand how their own disciplinary research connects to research in other disciplines less familiar to them in terms of concepts and methods (Klein 2005; Repko and Szostak 2020; Friis et al. 2023). Creating such understanding is an important precondition for successful knowledge integration, a core ambition of inter- and transdisciplinary research that seeks to contribute to solving real-world problems (Boon and Van Baalen 2018; Pohl et al. 2021; Deutsch et al. 2025).

In this paper, we explore how individual researchers from different disciplines can integrate their work in the context of an interdisciplinary research project, i.e., a joint effort by researchers from different academic disciplines to address a common research question. We present a typology of interdisciplinary collaborations that shall support the design and implementation of such collaborations at the project level. The typology shall systematize the thinking about interdisciplinary collaborations at different stages of the research process as a precondition for integration. Notably, it can help researchers understand the ways in which disciplinary research can be combined at different stages of a project to achieve interdisciplinary integration in terms of processes and outputs (O’Rourke et al. 2016; Pohl et al. 2021; Hoffmann et al. 2022a, b). To achieve these goals, we developed the typology drawing on insights from literature on research collaborations and the research process as well as on structured reflection on our own interdisciplinary research experience. We use the case of an interdisciplinary project on sustainable transformation of pesticide governance that we were involved in to show how the typology can serve as one starting point for critical reflection about interdisciplinary research design and implementation. Such reflection also generates lessons for knowledge integration in other inter- and transdisciplinary projects (cf. Black et al. 2023; Deutsch et al. 2021, 2024; Hoffmann et al. 2017), a form of research on the rise in past decades (Van Noorden 2015).

The remainder of the paper proceeds as follows. First, we describe the process of typology development and introduce the case context that informed this process. Second, we present the typology of interdisciplinary collaborations and situate it in the literature on interdisciplinary research, research design, and collaboration. Third, we illustrate the typology with examples from our interdisciplinary project on pesticide policy and practice and highlight successes and challenges in interdisciplinary collaboration. Fourth, we discuss the value and limitations of the typology and reflect on related lessons from our interdisciplinary collaboration process. Fifth, we conclude on how the typology can strengthen interdisciplinary collaborations in research addressing complex societal problems.

## Research design

### Process of typology development

We developed the typology of interdisciplinary collaborations through an iterative process, in which we worked back and forth between literature and structured reflection on our own interdisciplinary research experience. The basis for this process was our shared pragmatist assumption that plural epistemologies from different disciplines can be combined to create a more holistic understanding of sustainability challenges and to inform actions to tackle them (Johnson and Onwuegbuzie 2004; Caniglia et al. 2021). The iterative process was sparked by our joint desire to learn more about the approaches and research methods that each of us was using. This desire led us to reflect on potentials for combining them in an interdisciplinary project we have been involved in. To this end, we held five in-person workshops and 17 online exchange meetings over a span of three-and-a-half years (2021–2024). The workshops and exchanges were led and documented by the first author, who served as integration expert in the project (Hoffmann et al. 2022a). The process started as an open-ended experience exchange and turned into a more structured work process focused on conceptualizing different interdisciplinary collaborations.

In our interactions, we transitioned through the stages of “(1) comparing disciplines, (2) understanding disciplines, and (3) thinking between disciplines” (Kluger and Bartzke 2020), which are typical for interdisciplinary research processes. We began by introducing each other to the approaches and research methods we were using, examining their similarities and differences. This broadened our collective understanding of methodologies and increased awareness about the strengths and limitations of our own approaches. We then started to discuss how different elements of our approaches could potentially contribute to informing, supporting, and reflecting on interdisciplinary collaborations (cf. Black et al. 2023). In the discussion, which considered our different disciplinary perspectives, we discovered that the way in which we collaborated in our interdisciplinary research project involved very different stages of the research process and that this deserved more attention. As a result, we embarked on developing a novel typology of interdisciplinary collaborations.

The development of the typology itself rested on two interrelated pillars. The first one was a review of the literature on interdisciplinary research, research design, and collaboration. In this review, we discovered that while there were works on interdisciplinary iterations and mixed-method designs (e.g., Tobi and Kampen 2018; Kinnebrew et al. 2021; Grace et al. 2021), little work existed on connecting interdisciplinary collaborations to different stages of the research process. The other pillar was a structured reflection on the interdisciplinary collaborations in our own research project, which also helped us to discover what was missing in the literature. Once we had identified the gap, two of us developed a preliminary typology of interdisciplinary collaborations and, based on it, all of us then prepared systematic descriptions of collaborations in our project. In several iterations, we refined the typology further and sharpened the empirical illustrations to obtain a coherent reflection on different interdisciplinary collaborations. This work process, and the personal relations we built through it, reinforced the interdisciplinary collaborations we describe.

In addition, our work process fulfilled an important function in facilitating exchange about interdisciplinary knowledge integration. Again, our exchange discussed own experiences against the backdrop of existing literature, notably on integrative leadership (Hoffmann et al. 2022b; Deutsch et al. 2024) and on conditions for successful knowledge integration on individual, team, and institutional level (Deutsch et al. 2025).

### Case context

The context for our work was the interdisciplinary research project “Evidence-based Transformation in Pesticide Governance” (Trapego, 2021–2025).^1^ The Trapego project examined pesticide risk reduction in agri-food systems that, because of their complexity, warrant interdisciplinary analysis. Scholars have conducted inter- and transdisciplinary research on different aspects of agri-food systems, including food security (Acevedo et al. 2018), farm investments (Akimowicz et al. 2018), farmers’ seed demand (Almekinders et al. 2019), climate change adaptation of farmers (Feola et al. 2015), and foresight regarding agri-food system developments (Huan-Niemi et al. 2016). Pesticide risk reduction has received less interdisciplinary attention, even though the inherent trade-offs between economic, social, and environmental sustainability objectives make such a perspective particularly relevant (Möhring et al. 2020; Hofmann et al. 2023).

More specifically, the Trapego project explored the potential for an evidence-informed, sustainable transformation of agricultural pesticide governance and use in Switzerland (Ingold et al. 2024). The project’s academic contribution is to understand the role of different types and sources of evidence for policy and practice decisions (Hofmann et al. 2023). Its societal contribution is the identification of policy and practice options for reducing environmental and human health risks of synthetic pesticides (Ingold et al. 2024). Given this focus, insights on interdisciplinary collaboration in the Trapego project can be informative for other projects in sustainability transformation research.

Researchers in the Trapego project have integrated concepts, methods, and data from seven disciplines and research fields (cf. Porter 2006, p. 189). The mix has comprised natural, health, and social sciences, including agronomy, agricultural economics, decision analysis, environmental sciences, political science, public health, and inter- and transdisciplinary studies. The project, funded under a program for interdisciplinary research, has involved 18 researchers from five Swiss research institutions. As of 2023, the project team consisted of nine senior researchers (professors and group leaders) and nine early-career researchers (postdocs, PhD candidates, and research assistants). All authors of this paper have belonged to the group of early-career researchers. While we had not been part of designing the project, we have specified and implemented the planned interdisciplinary collaborations and have developed new collaborations throughout the project lifetime. The project’s interdisciplinary orientation was strong from the outset, with one of the five work packages focusing on science integration and another one focusing on synthesis (Ingold et al. 2024). One goal of the science integration work package was to reflect on lessons learned about interdisciplinary collaboration. While the project also had transdisciplinary elements that integrated perspectives of scientists and stakeholders, we focus here on our approaches to and learnings from a set of interdisciplinary collaborations.

**Fig. 1:**
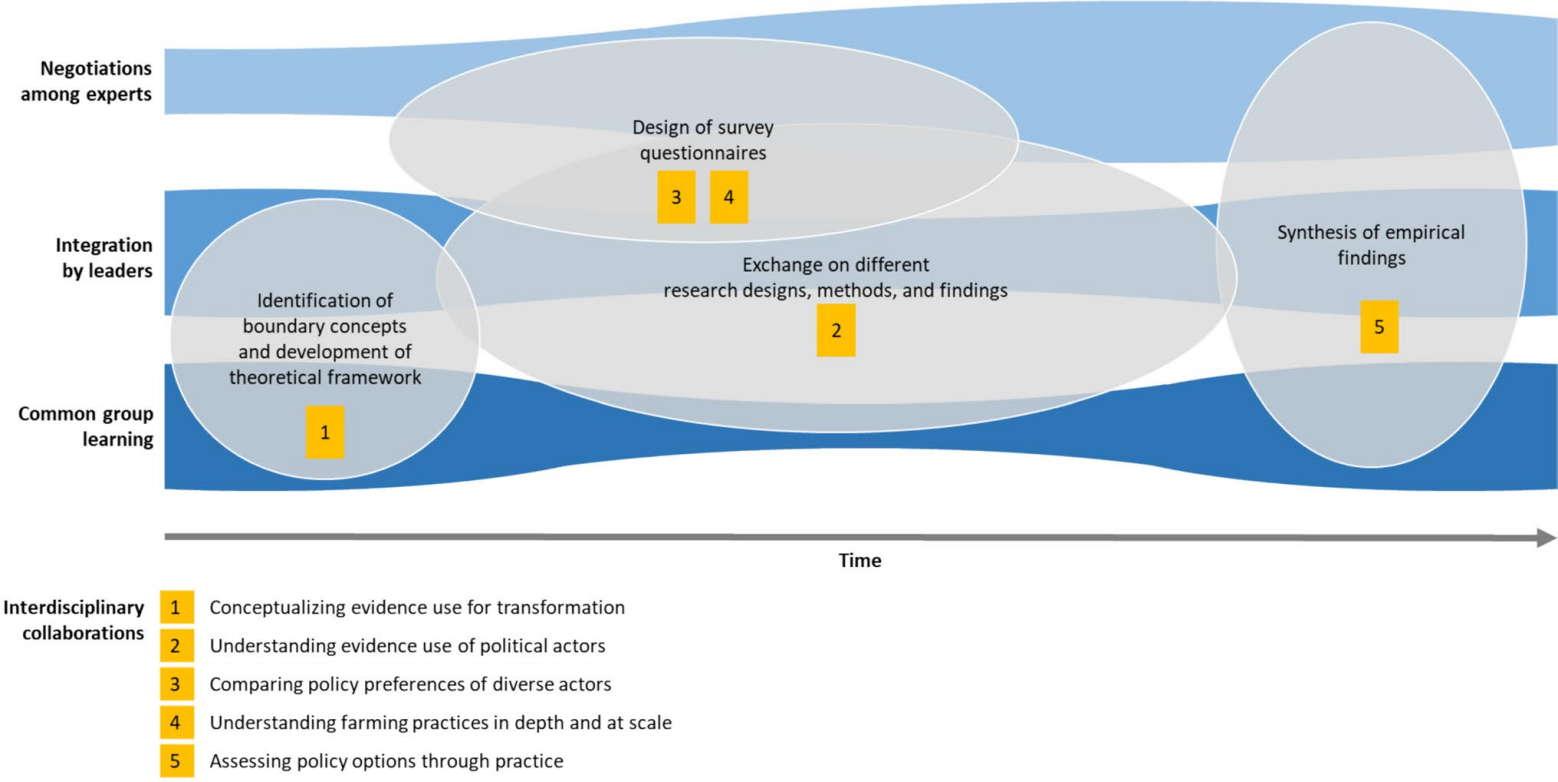
Stylized process of interdisciplinary boundary work in the Trapego research project. The figure shows the varying intensity of different procedures of integration over the project duration (blue streams), the broad boundary concepts and objects (gray bubbles; boundary settings not depicted), and the concrete interdisciplinary collaborations that emerged from them (yellow boxes). Source: authors

The interdisciplinary collaborations in the Trapego project emerged from a process featuring different procedures of integration. The procedures included common group learning, negotiations among experts, and integration by leader(s) (Hoffmann et al. 2017). The intensity of these procedures varied over the course of the project (Fig. 1). Moreover, in the design of the project, senior researchers determined that answering the overarching question in an integrated way would require boundary work in terms of concepts, objects, and settings (Star and Griesemer 1989; Mollinga 2010; Pohl et al. 2019). For boundary concepts, the focus was on developing a glossary of key concepts and a joint theoretical framework (Bergmann et al. 2013, chap. II.A) that helped to conceptualize evidence use in pesticide governance. Boundary objects comprised the design of coordinated stakeholder and farmer surveys, the recurring exchange about research design, methods of analysis, and findings, and the empirical synthesis of findings (Fig. 1). These boundary objects were instrumental in initiating interdisciplinary collaborations on the preferences and evidence use of actors as well as on farming practices and policy options. Boundary settings included dedicated resources for science integration, including the assignment of two integration experts who led, managed, monitored, assessed, and accompanied the integration process (Hoffmann et al. 2022a, p. 3). Throughout the project, different team-level meeting formats, including retreats, workshops, survey team meetings, and meetings of early-career researchers, served to update the team on research progress and—through tools such as research marketplace (Stauffacher 2020)—identify potentials for collaboration. Annual meetings with societal stakeholders, including national and subnational public administration as well as farmer and environmental interests, provided a sounding board for the project’s approach and findings. Numerous bilateral exchanges among researchers increasingly complemented team-level interactions. Common group learning and integration by leaders were essential for the conceptualization of evidence use and inspired or supported the other interdisciplinary collaborations, most of which, however, were implemented through negotiations among experts (Fig. 1).

## Types of interdisciplinary research collaborations

This section presents the typology of interdisciplinary collaborations we developed in the process and case context described above. To recall, we were interested in how scholars from different disciplinary backgrounds can integrate their research within an interdisciplinary project. Interdisciplinary integration refers to a constructive and open-ended “process of combining a wide range of perspectives from different disciplines” (Hoffmann et al. 2022a, p. 2) that leads to integrated outputs, such as synthesis reports (O’Rourke et al. 2016; Pohl et al. 2021; Hoffmann et al. 2022b). It can occur stand-alone or as integral part of transdisciplinary research. Research crossing disciplinary boundaries has been attributed manifold benefits in academic knowledge production (Nissani 1997) and in relation to society, including its ability to disentangle complex societal challenges (Kelly et al. 2019) and its greater societal visibility (D’Este and Robinson-García 2023). In view of these benefits, the question of how researchers can bring together different disciplinary perspectives in a joint effort has long been a topic of interest in collaborative research. However, much of the literature remains theoretical and lacks an answer to the question of how interdisciplinary collaboration can actually be implemented (Newman 2024). To fill this gap, we want to propose a typology that is conducive to the realization of interdisciplinary collaborations. We achieve this by linking the typology to the stages that are completed, in one way or another, in every empirical research process.

By research process, we mean a series of activities undertaken to systematically gather, analyze, and interpret data or information to answer a specific research question. In empirical studies, i.e., studies that rely on direct observations and measurements to answer a research question, the research process typically involves a number of stages (Kumar 2018), summarized in Table 1. While ideally the stages are sequential, it is often the case that, in practice, stages are worked on in parallel or that decisions from earlier stages are revised. In addition, we consider that distinct interdisciplinary research activities are possible at each stage.

**Table 1:** Ideal–typical stages in the research process*.* Source: Authors; stages and disciplinary research activities adapted from Kumar (2018)

No.	Stage	Disciplinary research activities	Interdisciplinary research activities
1	Research question	Formulating the guiding question that the disciplinary study seeks to address	Formulating overarching research question that bridges disciplinary sub-questions
2	Theoretical framework	Bringing together theoretical concepts, expectations, and hypotheses to form the theoretical framework of the study	Identifying boundary concepts and developing a joint theoretical framework with a set of hypotheses and expectations shared across disciplinary studies and/or using theories from different disciplines in complementary ways
3	Research design	Defining the strategy and tasks necessary to answer the research question, including decisions about: (a) research methodology (qualitative, quantitative, mixed methods), (b) case selection (population, sample, etc.), (c) methods used for data collection (survey, interviews, document analysis, etc.), and (d) analysis (social network analysis, regression models, etc.)	Using integrative methods (give-and-take matrix, theory of change, etc.), harmonizing variable operationalization and measurement instruments across disciplinary studies (e.g., module with harmonized survey/interview questions), and combining different analytical methods
4	Data collection	Actual gathering of information/data using predetermined methods such as interviews, surveys, or text analysis	Jointly gathering data for different disciplinary studies (e.g., joint expert interviews) and exploiting synergies in data collection (e.g., recruitment of survey participants)
5	Analysis/results	Carrying out the operations necessary to transform the data collected into meaningful information (i.e., results) that can be used to answer the research question. This may involve techniques such as regression analysis or social network analysis	Jointly analyzing data gathered in different disciplinary studies, e.g., of answers to harmonized interview questions from different surveys or by exploiting links developed between analytical methods
6	Discussion/conclusions	Drawing conclusions by interpreting, comparing, and critically evaluating findings and discussing them in relation to other findings and existing theories	Putting into perspective findings from different disciplinary studies and identifying connections between them that go beyond the scope of individual studies
7	Dissemination	Summarizing and communicating the results of the study, often in the form of a written paper, to share the knowledge generated	Synthesizing of related results from different disciplinary studies (including development of common language or translation into different disciplinary languages) and making synthesis available to the target audience (e.g., practitioners)

Among these stages, research design (no. 3) has received particular attention regarding the possibility to combine different methods. Proponents of mixed-method research argue that combining qualitative and quantitative methods can produce results that serve practical needs and, in a pragmatist research tradition, contribute to finding “tentative truths” (Johnson and Onwuegbuzie 2004; Johnson et al. 2007). Mixed methods are considered a promising approach for studying transformations in sustainability science (Wehrden et al. 2017) and have become more frequent in interdisciplinary fields, such as rural studies (Strijker et al. 2020). Recently, reviewing publications in conservation science, Kinnebrew et al. (2021) proposed a typology that distinguishes interdisciplinary mixed-method approaches according to the relationship of method objectives (nested or parallel) and the extent of integration (none, uni-, or bidirectional). Thus, method combinations can be an important element in interdisciplinary research collaborations.

Notwithstanding the importance of discussions about methods, we assume that research collaboration happens when two or more individuals work together *at any stage* of the research process to make another stage possible. This definition is based on the more general definition proposed by Katz and Martin in their seminal paper: “‘research collaboration’ could be defined as researchers working together to achieve the common goal of producing new scientific knowledge” (Katz and Martin 1997, p. 7). Based on this definition, research collaborations have been distinguished according to different aspects, such as collaborator characteristics (e.g., Hayat and Lyons 2017), motivations (e.g., Bozeman and Corley 2004), organizational structures (e.g., Chen et al. 2015), and division of labor (e.g., Laudel 2001; Morrison et al. 2003; D’Ippolito and Rüling 2019). While most of this literature on collaboration focuses on outputs, especially scientific publications, we argue that not all collaborations are reflected in jointly published outputs. A more comprehensive understanding of research collaborations thus requires a broader perspective, considering all stages of the research process. Our typology considers this and complements the last aspect, the division of labor, by emphasizing how collaborators contribute at different stages of the research and how their inputs shape both the process and its outputs. In doing so, our typology also contributes to a broader understanding of collaborations to better account for the dynamics and mechanisms of interdisciplinary research projects.

**Fig. 2:**
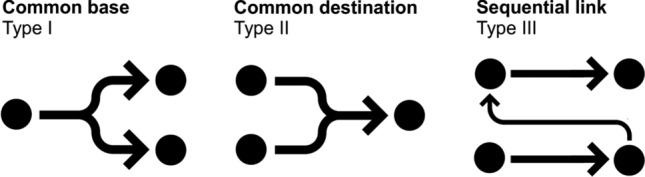
Types of interdisciplinary collaborations. Dots represent stages in the research processes. Source: authors

Based on our notion of the research process introduced above, we argue that there are three generic forms of collaborations that can be distinguished and that could start and end at any stage of the research process (Fig. 2). We refer to them as (I) common base, (II) common destination, and (III) sequential link. The first type, common base, describes a form of collaboration in which collaborators work together in the research process up to stage *s*, continuing with separate activities at stage *s* + 1. The second type, common destination, refers to a form of collaboration in which the collaborators work separately in the research process up to stage *s* and then jointly from stage *s* + 1 onward. The final type, sequential link, denotes a collaboration in which the output of stage *s* of a study A subsequently informs stage *s* − *x* of study B (where *x* can be ≥ 0). Apart from this link, the research processes of the two studies remain separate, i.e., study A and study B do not share any of the stages of the research process.

A particularity of our typology is that we do not understand interdisciplinary research projects as unified endeavors with a single research design and synthesis (Tobi and Kampen 2018). Rather, we conceive of interdisciplinary projects as a collection of separately designed collaborations that contribute to answering an overarching research question and can be synthesized in multiple ways. As the three forms of collaborations are ideal types, it is possible that, in practice, researchers combine them. Also, note that the stages in our typology refer to concrete research activities (e.g., running a model) and not to the preceding communication between collaborators that is necessary to carry them out (Wöhlert 2020).

The proposed typology supports interdisciplinary collaborations through strengthening researchers’ critical reflexivity. Reflexivity refers to “team members’ ability to reflect on and improve their own and the team’s knowledge, strategy, and processes” (National Research Council 2015, p. 77). Beyond this, critical reflexivity entails that researchers are able to recognize how their own methods of knowledge creation are situated in the diversity of the sciences (Halffman 2019) and of plural knowledge systems more broadly (Tengö et al. 2014). Greater clarity about researchers’ positionality helps to makes explicit how researchers’ subjective orientations may shape problem and solution framing (Lazurko et al. 2024), data collection, analysis, and the conclusions drawn from it (Berger 2015; Koot et al. 2023). Beyond recognizing subjective orientations, critical reflexivity also supports consciousness and identification of epistemological and ontological differences between involved disciplines (Danermark 2019; Horn et al. 2022b; Vladova et al. 2024). To navigate these differences, researchers need to understand how knowledge is produced in other disciplines (Boon and Van Baalen 2018), combine competencies in epistemic stability and adaptability (Horn et al. 2022a), and, ideally, develop a common team philosophy (Danermark 2019). The typology we put forward makes explicit at which stages of the research process different disciplines remain separate or come together and, thus, where epistemic stability or adaptability are needed—elements that can inform a common team philosophy.

Structured thinking about interdisciplinary collaborations also supports knowledge integration, which scholars have analyzed from different angles. Some accounts focus on attributes of integration, such as its dimensions (Pohl et al. 2021), level (Siedlok and Hibbert 2014), and change over time (Edelenbos et al. 2017). Other analyses are structured around additional concepts, such as organizational project areas (Lanier et al. 2018), structural levels (Deutsch et al. 2025), principles for leading, learning, and synthesizing (Hoffmann et al. 2022b), problem framing and aspirations (Cairns et al. 2020), synthesis procedures (Hoffmann et al. 2017), or specific methods (Deutsch et al. 2021). Focusing on types of interdisciplinary collaborations at different research stages, as we do here, can add a new angle to scholarly reflection. Especially, the proposed typology can encourage individual researchers to reflect on their position in and contributions to the knowledge integration process and on the challenges encountered therein.

## Empirical illustration: interdisciplinary collaborations on pesticide risk reduction

In the following sub-sections, we illustrate the typology by describing, in a structured way, five interdisciplinary collaborations we developed as researchers in the Trapego project (see Fig. 2 and Table 2). Each sub-section summarizes what the collaboration was about, how it was implemented, what opportunities and challenges it entailed, and how it contributed to the overall project goal of assessing potentials for an evidence-based transformation of pesticide governance in Switzerland. As the collaborations we describe here informed the typology development, their description has an illustrative purpose and cannot validate the typology across different project contexts. Nevertheless, we refer to these illustrations as “empirical” because our descriptions of the collaborations follow a harmonized structure, which supports abstracting from personal experience and facilitates cross-comparisons.

**Table 2:** Overview of interdisciplinary collaborations in the Trapego project. Source: Authors

Example	Type	Stages in the research process	Involved disciplines	Explanation and research contribution
(1) Conceptualizing evidence use for transformation	Common base (I)	Joint theoretical framework → separate research design	Agronomy/plant protection, agricultural economics, decision analysis, environmental sciences, political science, public health/epidemiology, and inter- and transdisciplinary studies	Development of conceptual framework, including glossary of key concepts, as basis for research into actor motivations, barriers, and types of evidence use in pesticide governance
(2) Understanding evidence use of political actors	Common destination (II)	Separate data collection → joint analysis	Decision analysis and political science	Combination of data from elite survey and media content analysis to understand evidence use in pesticide discourse in connection with political positions through social network analysis
(3) Comparing policy preferences of diverse actors	Common base (I) + common destination (II)	Joint research design → separate data collection → joint analysis	Agricultural economics, public health/epidemiology, and political science	Harmonized design and joint analysis of different surveys with political elite, farmers, and consumers to compare their support for transformation through new pesticide policies
(4) Understanding farming practices in depth and at scale	Sequential link (III)	Data collection and analysis → subsequent research design and data collection	Agronomy/plant protection and agricultural economics	In-person farmer interviews as an input to designing a relevant large-scale survey on farming practices of apple growers to assess pesticide risk reduction potential at farm level
(5) Assessing policy options through practice	Sequential link (III)	Data collection and analysis → subsequent research design and data collection	Agronomy/plant protection and decision analysis	Sustainability analysis of real-life farm data as basis for predicting effects of policy options on farming practices and pesticide risk reduction in multi-criteria decision analysis

### Example 1: conceptualizing evidence use for transformation

In this common base collaboration (type I), researchers from all seven disciplines and fields in the project jointly developed a conceptual framework as starting point for their varied research into evidence-based transformation. The framework development combined insights from existing literature on evidence use and sustainable transformation with the project team’s disciplinary observations on pesticide policy and practice. We theorized how different actor motivations shape, and potentially hinder, evidence use in pesticide governance and how this affects the potential for sustainable transformation (Hofmann et al. 2023). Thus, the collaboration centered on building a joint theoretical framework as basis for separate disciplinary (but conceptually connected) research designs and other interdisciplinary collaborations.

The collaboration took an abductive form that linked theory to empirics, and vice versa. The theoretical point of departure was an existing framework on links between pesticide policy and practice (Möhring et al. 2020) and a list of key concepts compiled by all project members. We then heavily modified the initial framework by adding key concepts from our list, such as evidence, actor motivations, and sustainable transformation. From this, we combined different stages of evidence use and actor motivations to derive barriers to an evidence-based sustainable transformation. We probed the plausibility of these barriers by drawing on empirical examples from scientific literature and our own knowledge of pesticide policy and practice in the Global North (cf. Hofmann et al. 2023). As this work progressed, we incorporated further concepts, such as scientific and experiential as well as problem- and solution-oriented evidence (Fazey et al. 2006; Raymond et al. 2010; Caniglia et al. 2017). Subsequently, project team members used elements from the conceptual framework in their disciplinary research designs or referred back to it in their analyses and discussions of results.

The collaboration, which had been outlined in the project proposal, involved various iterative interactions. Common group learning in workshops was used to brainstorm concepts, discuss a draft framework, and validate it. An early-career integration expert prepared these stages by leading the theoretical development and connecting it to literature on evidence use from different fields. To discuss questions arising in this work, the integration expert drew on quick iterative cycles with a core team composed of three senior researchers with natural and social science backgrounds. Additionally, bilateral exchanges between the integration expert and contributors solved open questions about the fit of the framework and empirical examples. Epistemological challenges encountered in the process included finding a common conceptual language suitable for all involved researchers and handling diverging views on some key concepts. Most of these differences were bridged successfully by raising the conceptual framework to a rather general level, abstracting from disciplinary epistemologies, and managing expectations about the level of detail accordingly. In a few cases, however, the research team had to agree to disagree in order to move forward. A practical implication for the project was that some integrative potentials remained unexploited for the benefit of pursuing more feasible disciplinary outputs.

The interdisciplinary collaboration helped to theorize a broad range of potential barriers to evidence use in pesticide policy and practice. The focus on barriers rooted in different actor motivations and evidence use stages was broad enough for many disciplines to connect to it. Its empirical illustration made the framework tangible for uptake in subsequent research. Most importantly, the conceptual framework structured the analytical space related to an evidence-based transformation in pesticide governance. This prepared the ground for research into different policy and practice aspects and for the interdisciplinary collaborations reported below.

### Example 2: understanding evidence use of political actors

In this common destination collaboration (type II), researchers from decision analysis and political science worked together to investigate how political actors gather information and which aspects of the acquired knowledge they then use as evidence in public discourse in connection with their political position (Reber and Wiget 2024). To this end, we combined data from an elite survey^2^ among Swiss policymakers and stakeholders and a manual content analysis of media documents related to pesticide use in Switzerland. The combined dataset allowed us to draw conclusions about the strategies of political actors when it comes to knowledge acquisition and the subsequent selection of key pieces of information used in their public communication. Thus, the merger of the research processes occurred at the analysis stage.

The collaboration exploited synergies that emerged from separate data collections. The elite survey allowed us to directly ask key political actors in Switzerland how they inform themselves about the ongoing domestic political process to reduce pesticide risks and about technical matters related to pesticide use. We asked them, for example, what type of sources (news media, research articles, personal contacts, etc.) they used to consolidate their opinions. The content analysis of media documents allowed us to record which sources actors of the same type or position then refer to in Swiss public discourse on pesticides to support their arguments. The availability of input and output data allowed us to compare the sources that the political actors either choose or neglect when they decide on their public communication strategy.

Although this is a type II collaboration, it was critical for its success that the two instruments used for data collection—survey questionnaire and content analysis codebook—were designed with the collaboration in mind and harmonized before data collection. This concerns both the types of data collected (i.e., the variables) and the way in which they were collected (i.e., the concepts and categories used). More specifically, it was necessary to find a common understanding of factual knowledge and its sources (i.e., who qualifies as knowledge producer and broker) in the development of data collection instruments. Furthermore, it was crucial to coordinate the collection of actor information to allow for subsequent merging of the data (e.g., actor type). A challenge we faced was that once we started collecting data, it became clear that the content analysis codebook needed to be adapted. Not all variables could be collected at the level of detail originally hoped for, which made it more difficult to draw conclusions from the two datasets (e.g., about political positions). We met this challenge by shifting the focus from the individual actor level to the aggregate level of actor types, sacrificing resolution and the goal of modeling evidence use and, instead, permitting only descriptive analysis.

Nevertheless, the results of this collaboration showed that, to consolidate their position, political actors generally use many sources that provide them with very different types of knowledge. In their public communication, however, they tend to rely on only a few pieces of information to substantiate their arguments. This shows that the use of evidence in public communication is indeed deliberate and tactical (Reber et al. under review). In addition, media actors play an important role as gatekeepers who ultimately decide what knowledge is disseminated to the wider public (Reber and Wiget 2024). In the context of the Trapego project, this helped to understand which evidence sources and pathways contribute to a sustainable transformation of Swiss agriculture and where barriers for knowledge uptake by policymakers exist.

### Example 3: comparing policy preferences of diverse actors

In this combined common base and common destination collaboration (types I + II), agricultural economists, health scientists, and political scientists worked together to investigate policy preferences of farmers, consumers, and the political elite. We studied how preferences of individual actors differed and whether they were congruent with their representative organizations (e.g., farmers and Farmers’ Union) for objectives and policies related to agricultural pesticide use in Switzerland. Data was collected through four different surveys (two for different farmer populations, one for consumers, and one for political elite). The interdisciplinary collaboration occurred before and after disciplinary data collection, notably in research design (common base) and analysis and discussion (common destination).

The collaboration harmonized elicitation methods across surveys to facilitate joint analysis for different actor groups. The focus was on ensuring the same methodical consideration of policy objectives within different online surveys. To obtain policy preferences, actors were asked about their support for different measures (e.g., taxation, regulation, and subsidies) and their preferences for who should regulate pesticides (market, state, etc.). Additionally, in three surveys, we elicited preferences regarding certain policy objectives (e.g., human health, environment, and agro-economy) to gain a better understanding of potential differences in policy preferences between actors and their organizations. The surveys were tailored to targeted actor groups (e.g., by adapting the language/terminology to farmers and policy actors in the survey questions) and to disciplinary considerations (e.g., how to elicit specific preferences). Despite these adjustments, collaboration in study design ensured the collection of comparable data about policy preferences, which allowed for a joint data analysis and discussion (e.g., Zachmann et al. 2023). We were able to compare different preferences without different elicitation methods influencing the data across actor groups. However, this required balancing data collection for disciplinary research and collaborative purposes. Too tailored questions would have been unsuitable for collaboration purposes, whereas too general questions would have had limited value for disciplinary research. Detailed data was aggregated to broader measures, allowing collaborative as well as disciplinary analyses. Nevertheless, not all preferences could be covered in every survey, meaning that comparison had to be limited to core questions of joint interest. Further challenges arose regarding the timing and extent of surveys, as teams participating in the collaboration had different constraints regarding survey scope and timelines (e.g., researchers working on farmer’s health needed to approach them off-season time). Thus, teams implementing their survey later followed the previous survey’s design and question formulation or were invited to co-design follow-up questionnaires. In the necessary selection of survey items, those relevant for purposes of interdisciplinary synthesis were prioritized.

The collaboration emerged from regular exchanges between scientists in the survey design process. In discussions on policy preferences in different actor groups, we recognized that coordinating our separately planned data collections could shed light on the congruence of individual and organizational preferences. We then compared potential approaches to preference elicitation across agricultural economics, health sciences, political sciences, and decision analysis. Next, we discussed how these approaches could be adjusted for harmonization across actor groups. Mutual adjustments resulted in harmonized questions with the same answer scales and response options relevant to targeted actors. As the survey on farmers’ health was implemented later than the other surveys, improvements based on feedback from previous surveys could be implemented (e.g., deletion of redundant policy objectives). Throughout the survey design process, epistemic adaptability in the form of openness and critical reflection were essential to select the most appropriate method and to avoid bias towards the methods usually used in each researcher’s field (favoritism).

Investigating preferences for new policies of individuals involved in policymaking or affected by policies is an important information towards transformation, especially in democratic systems (Dermont et al. 2017; Peckham et al. 2022). The collaboration provided such information, going beyond the capacity of single disciplines. It helped to compare preferences for trade-offs among conflicting policy objectives and policy preferences of actors and their organizations, allowing us to investigate the political feasibility of new policies and related practices. Importantly, the two farmer surveys increased the number and types of farmers reached, as the agro-economic survey focused on apple producers, while the health survey included all farming profiles. These insights into support for new agricultural policies were an important element in assessing the transformation potential in Swiss pesticide governance.

### Example 4: understanding farming practices in depth and at scale

In this sequential link collaboration (type III), agricultural economists and plant protection specialists in agronomy joined forces to investigate the potential for reducing pesticide risks in Swiss apple farming. The collaboration built on interviews with farmers to gain in-depth understanding of their plant protection strategy throughout the season. Insights from these interviews, including data on the farm’s whole pesticide record, farm and field equipment, apple varieties grown, and farm management, helped to design a survey reaching more farmers with more diverse farming profiles across Switzerland (Zachmann et al. 2023). This sequence facilitated a thorough exploration of pest management practices and pesticide use across different farming systems (e.g., conventional and organic).

The collaboration strategically combined small- and large-n research designs. In face-to-face interviews with farmers (*N* = 20), a plant protection specialist elicited detailed data on their pest management practices. Interviews were comprehensive in terms of production systems (conventional and organic), pesticide applications (fungicides, insecticides, and herbicides), and farming aspects covered (e.g., farm and field characterization, pesticide and fertilizer records, and costs and benefits of apple crop and pest management). Agricultural economists drew on this information to develop an online survey (*N* = 245) on apple farming, encompassing a broad range of farm-level agronomic practices. This approach had several benefits: it enriched online research with real-world insights; helped to streamline the survey with a view to shorten response time; tailored the questionnaire to the needs and interests of apple growers, potentially enhancing farmers’ trust and support; and allowed for survey pre-testing by initial interviewees, which helped adjusting questions to capture the nuances of pest management in apple farming.

The collaboration was implemented in a structured manner. First, processed data from the in-person interviews was shared to identify common themes and provided preliminary insights into the hypotheses underlying the large-scale survey. Second, researchers held regular meetings to facilitate in-depth discussions of common themes, leveraging extensive knowledge from the interviews. Third, collaborating researchers generated a list of questions for the survey, along with suggested answer options for some questions. The entire process involved iterative exchanges, allowing us to fine-tune and refine the content of the large-scale survey to ensure its effectiveness and relevance. Challenges arose in relation to data integration due to discipline-specific terminologies and coordination across disciplines. These challenges were addressed by extensive communication across disciplinary teams and agreeing common definitions.

The interdisciplinary sequence of mixed data collection methods yielded a comprehensive overview of current farming practices and factors influencing them. These insights provide evidence for policymakers about which pesticide practices are used and about potential focal points for policies to reduce pesticide risks—both being important for the transformation of Swiss pesticide governance. In particular, this overview helped to study pesticide use reduction potential at the farm level by zooming into cosmetic pesticide use aimed at increasing the visual quality of agricultural products and thus allowing the identification of pesticide risk reductions without compromising food security (Zachmann et al. 2024). Additionally, the collaboration mitigated potential data quality concerns, as information of survey questions was backed by insights from detailed interviews and thus contributed to more accurate answering of each question.

### Example 5: assessing policy options through practice

This sequential link collaboration (type III) among researchers from plant protection/agronomy and decision analysis explored farming practices and policy options for pesticide risk reduction in Switzerland. The collaboration consisted of agronomic case studies of crop protection practices on farm level and a multiple-criteria decision analysis (MCDA, Eisenführ et al. 2010) on policy options for pesticide risk reduction. The agronomic case studies developed an inventory of best and worst practices in sustainable crop management and pesticide use. The MCDA assessed the performance of policy options in relation to multiple objectives (environmental protection, agricultural production, etc.) and stakeholders’ preferences in relation to these objectives. Thereby, the results from agronomic case studies provided the basis for the construction of the MCDA.

The collaboration combined analytical methods and tools in innovative ways. The plant protection researcher used the assessment tool SMART (Sustainability Monitoring and Assessment Routine; Schader et al. 2016)^3^ to analyze agronomic data from face-to-face interviews with farmers. Several areas of farm management were evaluated based on international guidance (SAFA: Sustainability Assessment of Food and Agriculture Systems; FAO 2014), including financials, crop protection strategy, farm characteristics (e.g., size, crops, and farming system), and sociological context. Information on indicators from the SMART analysis aided decision analysts in defining the policy objectives and indicators in the MCDA. Furthermore, insights from farm interviews informed the consideration of policy options in the MCDA that could support sustainable crop protection practices. Besides inputs from SMART, the decision analysts drew on stakeholder workshops and expert interviews to identify policy objectives and indicators, elicit stakeholder preferences, and assess policy options (Gregory et al. 2012). Hence, interdisciplinary collaboration allowed for studying policy options in the context of current farming practice.

The collaboration required regular exchange of data and results between researchers conducting the SMART analysis and MCDA. The initial focus was on creating a mutual understanding of farming practices and the two analytical tools. Later, the focus shifted to constructing a joint approach on how to link policy options with farming practices. For instance, for the policy options considered in the MCDA, different farming practices were selected based on their potential for a sustainable transformation of crop protection as assessed in the agronomic case studies. In interviews jointly conducted by plant protection and decision analysis researchers, the effects of specific policies on these practices and on policy objectives were further discussed with experts. A challenge of the collaboration was that, while inclusion of SMART results into the MCDA yielded a richer analysis, it also increased the level of complexity and time needed. The need for abstraction in the policy-level analysis competed with the heterogeneity of farm-level decisions in response to policies (e.g., depending on the cultivated crops and farming system). The challenge was met through a regular exchange, in which different categories of sustainability objectives and agricultural practices were compiled from the detailed SMART analysis data, and then linked to policy objectives and instruments. The farm-level categorization served as a guide for developing an appropriate objectives hierarchy and realistic assumptions about the impact of policy instruments.

Evidence on farming practices is indispensable to inform policies and assess their potential effects on pesticide use. Without policy objectives and performance indicators rooted in practice and without an understanding of how farming practices and pesticide risk reduction policies interact, predicting the transformation potential of policy options is difficult (cf. Möhring et al. 2020, 2023; Hofmann et al. 2023). Our integrated assessment with transparent and comprehensive linkage of farming practices and pesticide policy options provided a good starting point for such predictions and can inform future discussions with stakeholders.

## Discussion

The typology we propose has both an internal and external value for interdisciplinary research projects. Internally, it can create a systematic understanding of how different disciplinary work is connected, a function typically fulfilled by structuring projects into work packages (Donaldson et al. 2010; König et al. 2013; Edelenbos et al. 2017; Lindvig and Hillersdal 2019). To foster integration, however, continuous thinking about connectedness beyond disciplinary silos seems more important than work packages. The latter may discourage unplanned interdisciplinary collaborations or, if structured along disciplinary lines, even hinder integration (cf. Djinlev et al. 2023, pp. 173–174). Furthermore, our illustrative empirical examples suggest it might be misleading to treat research as a disciplinary process that is complemented by interdisciplinary iterations outside of the main research process (Grace et al. 2021). Rather, the typology helps to develop interdisciplinary collaborations as an integral part of various research stages and to clarify their contribution to overarching research and societal impact goals. At the same time, by breaking integration into smaller parts, the typology mitigates potential fears of project members about the need for an all-encompassing synthesis in the end of the project. Externally, the typology of interdisciplinary collaborations makes the project design more transparent and comparable for researchers and stakeholders from outside the project. This can facilitate new collaborations across and beyond existing project consortia.

Despite these benefits, our typology also has limitations. A first limitation is its broad focus on interdisciplinary collaborations that cannot provide operative guidance on how to combine specific empirical research methods. To get there, our typology could be complemented with insights into combinations of qualitative and quantitative research from mixed-method literature (Johnson and Onwuegbuzie 2004; Johnson et al. 2007; Akimowicz et al. 2018; Kinnebrew et al. 2021; cf. Tobi and Kampen 2018). A second limitation is that our typology necessarily simplifies the variety of interdisciplinary collaborations. While we distinguish three ideal types, real-world collaborations might represent mixed types or go beyond the typology’s scope (Siedlok and Hibbert 2014; Pedersen 2016). A third limitation is that the typology applies to inter- rather than transdisciplinary research, which transgresses the boundary between academia and other societal sectors. If combined with knowledge coproduction principles (Lang et al. 2012; Norström et al. 2020), however, it could potentially also add to structured thinking about transdisciplinary collaborations, especially as interdisciplinary perspectives are often an integral component of these. A fourth limitation is that we developed and illustrated the typology in the context of a single research project. Future research could test and validate the typology in other project contexts to refine or add to the collaboration types we identified.

The typology has potential for generalization to interdisciplinary research in areas beyond agri-food. One such area are sustainability challenges characterized by complex interconnections between environmental, social, and economic aspects, such as climate change (Schipper et al. 2021), conservation and development on land (Hirsch and Brosius 2013), and ocean protection and use (Hofmann 2022). Other application areas are those featuring complex policy-practice interactions and pursuing evidence-informed decision-making, such as in education, health, and welfare (cf. Rickinson et al. 2021). To generalize our findings, however, more empirical testing is needed, especially as our case mainly served to illustrate the typology. Moreover, the distance between the disciplines was limited in our case, which facilitated interdisciplinary collaboration through greater epistemological and ontological proximity. Further research is needed to understand how collaborations between more distant disciplines can be fostered.

Insights from the case suggest lessons for knowledge integration in other interdisciplinary projects, which resonate with existing literature. To begin with, interdisciplinary collaborations require a mix of common group learning in the project team, integration by a leader, and negotiations among experts (Hoffmann et al. 2017). While joint work in the larger team (e.g., development of a conceptual framework) laid the foundations for the interdisciplinary collaborations in our case, smaller ad-hoc teams developed and implemented these collaborations. Furthermore, project proposals need to create conditions conducive to interdisciplinary collaborations (Lanier et al. 2018; Deutsch et al. 2025). In our case, conducive boundary settings (Mollinga 2010, pp. 6–7), such as establishment of an integration expert position and regular retreats, workshops, and meetings with all project members or in sub-groups (e.g., early-career researcher meetings), guaranteed dynamic and continuous collaboration. Additional factors that helped maintain interdisciplinary collaborations included open communication, diverse roles of researchers (Hofmann et al. 2025), and flexible adaptation of research design. At the same time, we recognize that the limited distance between our disciplines probably narrowed our problem framing and that most of us considered transformation potentials mainly within certain system boundaries. While this was conducive to knowledge integration across disciplines, it may have excluded other relevant perspectives, such as a more critical engagement with agro-chemical companies and the food system (e.g., Clapp 2018, 2021). Although the three types of collaboration described are equally valuable in theory, their specific applicability, benefits, and limitations clearly depend on the individual project context and the researchers involved.

The case also reveals trade-offs that interdisciplinary collaborations need to navigate. One trade-off is between the more disciplinary research interests of individual researchers and the common interdisciplinary research interest of the larger project. A coping strategy for researchers is to identify with the project through a value it carries for them, such as, in our case, venturing into areas outside one's own silo, changing one’s own mindset through systems thinking, and contributing to more sustainable agriculture. Practically, however, it may still prove challenging to reconcile the epistemic and ontological traditions as well as thought and work styles that researchers from different disciplines bring to an interdisciplinary project (Halfon and Sovacool 2023; Pohl et al. 2021; Siedlok and Hibbert 2014). Another trade-off concerns the different aims and requirements of interdisciplinary data collection and disciplinary publication of findings. Transparent communication of disciplinary expectations and early identification and negotiation of collaboration potentials can help overcome this trade-off. Yet, tensions may occur between the need to plan ahead (e.g., data collection) and the iterative, open-ended nature of interdisciplinary collaborations (cf. Hoffmann et al. 2022b). Coping with these trade-offs and tensions calls for project leaders and integration experts who act as mentors, facilitators, and mediators (König et al. 2013; Hoffmann et al. 2022a) as well as for high engagement of all team members (Deutsch et al. 2021).

Our results raise several new questions about interdisciplinary research. First, it seems important to explore how to document and measure the success of interdisciplinary collaborations within projects. This could provide a stronger and more fine-grained empirical base for evaluating the success and impact of interdisciplinary research (cf. Goring et al. 2014; Wang et al. 2015), as well as for giving due recognition to all contributors. Second, we wonder which factors can foster each of the three types of collaboration. For instance, future research could deepen knowledge about which funding conditions and project designs are most conducive in this respect (cf. Lyall et al. 2013; Szostak 2017). Third, regarding the typology, we would be curious to see whether future case studies corroborate the three collaboration types or discover additional types or other possible combinations.

## Conclusions

Interdisciplinary collaborations can be distinguished meaningfully regarding whether they (I) start from a common base, (II) have a common destination, or (III) represent a sequential link. This parsimonious typology underscores that interdisciplinary collaborations can unfold at different stages of the research process. We illustrated each type of collaboration with examples from an interdisciplinary research project on the transformation of pesticide governance. While the details of each collaboration may be specific to our project context, they illustrate the benefits and trade-offs of different types of interdisciplinary collaborations. Our findings are of interest to other interdisciplinary research projects and to transdisciplinary projects that build on interdisciplinary perspectives in three ways. First, the proposed typology can structure the thinking about interdisciplinary collaborations for project members and external partners. Second, we described a process for creating and maintaining collaborations across disciplinary boundaries that researchers can apply in other project contexts. Third, the typology can support reflection about interdisciplinary collaboration and potentials for further improving knowledge integration.

Our findings have implications for inter- and transdisciplinary research on sustainable transformation and other complex societal challenges. One implication is that such research needs a dedicated integration process supporting the formation, continuation, and harnessing of interdisciplinary collaborations (Lanier et al. 2018). Many of our collaborations evolved from learning processes in the project team or among experts (cf. Hoffmann et al. 2017). In this process, researchers identified and discussed boundary concepts and objects and regularly exchanged about their empirical work. The integration process also comprised team-level reflection aimed at “learning to collaborate while collaborating” (Freeth and Caniglia 2020, p. 247). To support this process, integration needs to receive sufficient resources in project funding, planning, and implementation (e.g., in terms of integration expert positions, budget, and time and commitment of all project members) (Hoffmann et al. 2022a). Another implication is that there is no uniform way of doing interdisciplinary research. Interdisciplinary collaborations take different forms and involve different research stages. This diversity allows for creativity in research (Szostak 2017) but can cause confusion as researchers might struggle to find their way. The typology provides orientation in interdisciplinary collaboration and could be combined with other inter- and transdisciplinary collaboration tools (e.g., give-and-take matrix; Stauffacher 2021).

Finally, our results underscore that interdisciplinary research is both valuable and challenging (cf. Guimarães et al. 2019). In our case, interdisciplinary collaborations greatly contributed to answering a research question about sustainable transformation that no single discipline alone could have answered comprehensively. Yet, collaborations had to deal with challenges, especially time limitations and disciplinary output requirements. As a group of early-career researchers, we observed that academic expectations for the performance of postdocs and PhD candidates are still strongly informed by quantitative disciplinary logics. Some of us experienced difficulties in engaging in interdisciplinary collaborations that emerged during the project on top of their pre-planned outputs or in getting recognition for their contributions to interdisciplinary collaborations. These challenges may undermine the motivation of early-career researchers to take part in interdisciplinary efforts. To foster integration, we suggest relaxing quantitative output expectations for early-career researchers in interdisciplinary projects and, instead, evaluating them on the quality of collaborations they developed and their contributions to common group learning and its varied outputs (cf. Goring et al. 2014). This, we think, can promote more interdisciplinary collaborations of all three types to better understand and tackle pressing societal problems.

## Data Availability

We do not analyse or generate any datasets, because our work proceeds within an experience-based approach. The details of our research design are described in the manuscript.

## Footnotes

1 For more information, see http://www.trapego.ch.

2 The “elite” are politically active actors who individually or collectively influence policy decisions because of their constitutional role or socio-political power (Hoffmann-Lange 2018).

3 See https://www.fibl.org/en/themes/smart-en/smart-method.
